# Effective connectivity during animacy perception – dynamic causal modelling of Human Connectome Project data

**DOI:** 10.1038/srep06240

**Published:** 2014-09-01

**Authors:** Hauke Hillebrandt, Karl J. Friston, Sarah-Jayne Blakemore

**Affiliations:** 1 Institute of Cognitive Neuroscience, University College London, London, WC1N 3AR, United Kingdom; 2 Moral Cognition Laboratory, Department of Psychology, Harvard University, Cambridge, MA, 02138, United States; 3 Wellcome Trust Centre for Neuroimaging, Institute of Neurology, University College London, WC1N 3BG, United Kingdom

## Abstract

Biological agents are the most complex systems humans have to model and predict. In predictive coding, high-level cortical areas inform sensory cortex about incoming sensory signals, a comparison between the predicted and actual sensory feedback is made, and information about unpredicted sensory information is passed forward to higher-level areas. Predictions about animate motion – relative to inanimate motion – should result in prediction error and increase signal passing from lower level sensory area MT+/V5, which is responsive to all motion, to higher-order posterior superior temporal sulcus (pSTS), which is selectively activated by animate motion. We tested this hypothesis by investigating effective connectivity in a large-scale fMRI dataset from the Human Connectome Project. 132 participants viewed animations of triangles that were designed to move in a way that appeared animate (moving intentionally), or inanimate (moving in a mechanical way). We found that forward connectivity from V5 to the pSTS increased, and inhibitory self-connection in the pSTS decreased, when viewing intentional motion versus inanimate motion. These prediction errors associated with animate motion may be the cause for increased attention to animate stimuli found in previous studies.

Humans are highly proficient at detecting animate movement in other agents, or noticing entities that act intentionally in the world, a capacity that has adaptive benefits for behaviour^1^. The attribution of agency is so automatic and irresistible that humans attribute agency even to simple two-dimensional shapes that appear to move in a self-propelled way^2^. Cues that trigger the perception of agency include non-Newtonian motion and sudden changes in motion direction and speed^3^. Animate motion is usually more complex, nonlinear and thus unpredictable than inanimate motion, because self-propulsion relies on hidden internal (intentional) causes of an agent^2^. In contrast, inanimate object motion of the kind humans encounter in their natural environment is generated by relatively invariant forces – such as a stone falling due to gravity, or a ball being hit by a snooker cue. Here, we tested whether these differences in predictability between animate and inanimate motion stimuli would be associated with differences in brain connectivity measures hypothesized to underlie sensory prediction errors.

While animate motion is generally less predictable for humans than inanimate motion^4^, not all forms of animate motion are objectively more unpredictable than inanimate motion. For instance, repetitive (stereotyped) behaviours, such as walking, are more predictable than some inanimate motion, because repeated exposure to the same stimulus leads to repetition priming and neural adaptation – an effect that has been attributed to increased predictability^5^,^6^. Inanimate Brownian motion is also more difficult to predict than animate motion trajectory patterns. However, humans are unlikely to have evolved to predict Brownian motion, and do not naturally attend to Brownian motion. In contrast, the prediction of animate motion is crucial for survival – and it has been suggested that the visual system has evolved to predict biological motion of other animals such as insects^7^. Moreover, only repetitive human biological motion, such as walking, is easy to model and predict. The trajectories of animals such as insects over extended timescales are hard to model and predict, and in practice, almost as difficult to predict as Brownian motion^8^. Due to this unpredictability, viewing of animate motion trajectories compared with inanimate motion should produce more salient or informative sensory prediction error signal (a mismatch between the model's prediction and the sensory input). In short, our prediction errors are much smaller when modelling the trajectories of an (inanimate) pool ball relative to an (animate) bug crawling over a pool table. Here, we test this hypothesis by analysing fMRI data from 132 participants from the Human Connectome Project (HCP)^10^ to quantify directed (effective) connectivity of the forward connection and the backward connection, which convey sensory prediction error signals and prediction signals respectively, during animate versus inanimate motion perception.

fMRI data were collected while participants viewed triangles designed to move in either an animate or inanimate way and Dynamic Causal Modelling (DCM) was used to model the induced neural responses. DCM estimates the experimental modulation of (intrinsic) self-connections or (extrinsic) forward and backwards connections between brain regions that are active during a particular task in a directional manner. We used a novel post-hoc model selection routine^10^,^11^ to investigate all possible dynamic causal models, and tested the hypothesis that the forward connection, which conveys sensory prediction error signals, is selectively more engaged than the top-down backward connection, when people view animate motion compared with inanimate, mechanical motion. We quantified the effective connectivity between only two regions. The first region was V5, the canonical motion sensitive area^12^, which is responsive to any type of visual motion (animate and inanimate). The second region was the pSTS, a region that is particularly highly activated when participants view animate (relative to mechanical) motion^13^. It is common practise in DCM to restrict the analysis to only a small number of co-activated regions, however, the connectivity between these regions can be mediated polysynaptically by other (excluded) regions^14^.

We investigated whether augmented pSTS responses during the perception of animacy are mediated by a selective increase in sensitivity of forward afferents from motion sensitive V5, or whether they reflect a non-specific increase in the pSTS excitability mediated by top-down effects from unmodelled higher level areas. We predicted both an increase in the forward effective connectivity from V5 to the pSTS and a decrease in the self- or intrinsic-connectivity within the pSTS. In addition, we predicted that forward connectivity would be modulated more strongly than backward connectivity, due to increased prediction error (and potentially the change in attentional selection and/or gain modulation that is associated with increased prediction errors, see^15^ for a discussion).

## Methods

### Participants

134 healthy adults were initially considered for DCM analysis (89 female; all but one participant were between 22-35 years old with a mean age of approximately 30.5 years, see Van Essen et al.^16^ for why reporting of exact ages would endanger anonymity of participants). Two participants were excluded: one because of missing onset files; another participant did not show activation in the one of the predicted areas during one of the sessions and thus could not be included in the DCM analysis (see *Volume of Interest Extraction section below*). Thus, data were analysed from 132 participants. The experiments were performed in accordance with relevant guidelines and regulations and all experimental protocol was approved by the Institutional Review Board (IRB) (IRB # 201204036; Title: ‘Mapping the Human Connectome: Structure, Function, and Heritability'). Written informed consent was obtained from all participants. Our data analysis was performed in accordance with ethical guidelines of the University College London ethics committee.

### fMRI data acquisition

See Ugurbil et al.^17^ for a detailed description of the HCP fMRI acquisition protocols. The following abbreviated overview is taken from Barch et al.^18^. Briefly, whole-brain EPI acquisitions were acquired with a 32 channel head coil on a modified 3 T Siemens Skyra with TR = 720 ms, TE = 33.1 ms, flip angle = 52°, BW = 2290 Hz/Px, in-plane FOV = 208 × 180 mm, 72 slices, 2.0 mm isotropic voxels, with a multi-band acceleration factor of 8^19^,^20^, as cited in ref. 18. Two runs of the task were acquired, one with a right-to-left and the other with a left-to-right phase encoding^18^.

### fMRI data preprocessing

We used the “minimally processed” Q2 release of the HCP data for this study (Functional Pipeline v2.0; Execution 1). These time series data were preprocessed using tools from FSL and FreeSurfer to implement gradient unwarping, motion correction, fieldmap-based EPI distortion correction, brain-boundary-based registration of EPI to structural T1-weighted scan, non-linear (FNIRT) registration into MNI152 space, and grand-mean intensity normalization^18^. See Glasser et al.^21^ for a detailed description of fMRI preprocessing of the HCP.

### Experimental design

**Figure 1:**
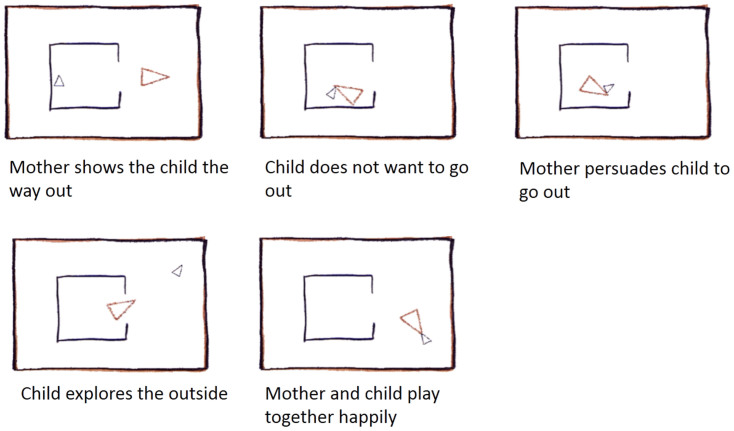
Example of “Theory of Mind” animation: The Big Triangle coaxing the reluctant Little Triangle to come out of an enclosure (participants do not see captions; stimuli and description adapted from^13^.)

The following abbreviated overview is taken from Barch et al.^18^. A well-validated task was used to probe animacy and agency detection. The stimuli have been shown to generate robust task-related activations that are reliable across participants in brain regions associated with social cognition (Castelli et al., 2000, Castelli et al., 2002, Wheatley et al., 2007 and White et al., 2011 as cited in ref. 19). Participants viewed short video clips (20 s) of objects (squares, circles, triangles) either interacting in some way (Animate motion), or moving mechanically (Inanimate motion)^18^. The basic visual characteristics in terms of shape, overall speed and orientation changes were matched between stimulus categories^13^. After each video clip, participants rated the video by choosing from three different options, depending on whether the objects contained a social interaction (an interaction in which the shapes appear to be taking each other's feelings and thoughts into account), Not Sure, or No interaction (i.e., there is no obvious interaction between the shapes and the movement appears random). Each of the two task sessions comprised 5 video blocks (2 Animate and 3 Inanimate in first session, 3 Animate and 2 Inanimate in the other session). Note that even though there were an unequal number of videos per conditions within each session, all our analyses took into account the data of both sessions at once, and thus our effects were not influenced by session specific effects. There were also 5 fixation blocks (15 s each); each video block was followed by a fixation block. Of note, the video clips were shortened to 20 s (the Castelli et al.^13^ clips were originally 40 s) by either splitting the videos in two or truncating them. A pilot study by Barch et al.^18^ confirmed that participants rated these shorter videos similarly. Figure 1 shows stills from an example of an Animate motion video.

### fMRI Data analysis

fMRI data were further analysed by us, using Statistical Parametric Mapping (SPM12b, www.fil.ion.ucl.ac.uk/spm). The 2 × 2 × 2 mm minimally preprocessed images were spatially smoothed with a 4-mm Gaussian kernel to increase the signal to noise ratio, while retaining sufficient anatomical acuity for extracting visual sensory areas. We did not slice time correct the data, nor did we later specify different acquisition times in the DCM model^22^, as simulated DCM data has been shown to cope well with slice timing differences of up to 1 s^22^ and our TR was 0.72 s. The time series were modelled with boxcar regressors based on two types of task block: Animate motion and Inanimate motion. In order to use the ‘All motion' contrast as a single input to the DCM, and the Animate – Inanimate motion contrast as a modulator of effective connectivity (and not use both animate and inanimate motion separately as inputs), we created appropriate parametric regressors^23^. These regressors were orthogonal to each other (the first regressor was All Motion – implicit baseline, and the second was Animate – Inanimate motion). In other words, the first regressor was non-specific motion effects, relative to baseline, while the second modelled animacy effects during motion. In addition, we included constant session effects. Appropriate stimulus functions were convolved with the canonical hemodynamic response function to form regressors for standard SPM analyses. Together with regressors representing residual movement-related artifacts and their derivatives, these regressors comprised the full (general linear) model (GLM) for each session. A group level ANOVA was performed to identify significant regional effects for the All Motion contrast and a contrast for Animate – Inanimate motion. All analysis scripts are available online (https://github.com/HaukeHillebrandt/SPM_connectome); this ensures the analyses reported below can be replicated and extended with the openly available HCP data (see discussion).

### Dynamic causal modelling

DCM estimates the experimental modulation of (intrinsic) self-connections or (extrinsic) forwards and backwards connections between brain regions that are active during a particular task in a directional manner. This enables one to infer whether experimental manipulations affect top-down, bottom-up influences or both. We used a novel post-hoc model selection routine^10^,^11^ to investigate all possible dynamic causal models, and tested the hypothesis that the forward connection, which convey sensory prediction error signals, are selectively more engaged than the top-down backward connection, when people view animate movement compared with inanimate movement. Specifically, we quantified the effective connectivity between V5, which is responsive to any type of motion (animate and inanimate) and the pSTS, which is selectively activated when participants view animate motion^13^.

### Specification of dynamic causal models

**Figure 2:**
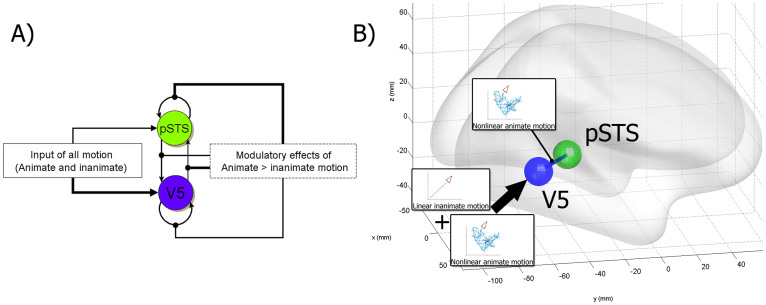
(A) The winning model: The full model was the winning model, with the highest evidence; in this model all connections were modulated by the Animate – Inanimate motion modulator. The driving input, the ‘All motion' contrast, entered into V5 and the pSTS. Wider lines represent stronger modulation or input relative to its comparison: V5 received more input (*Mean parameter estimate* = 0.96) than the pSTS (*Mean parameter estimate* = −0.06) and the Animate – Inanimate motion contrast modulated the forward connection from V5 to the pSTS significantly more strongly (*Mean parameter estimate* = 1.22) than the backward connection (*Mean parameter estimate* = 0.16) and the (inhibitory) self-connection of the pSTS (*Mean parameter estimate* = −0.19) less strongly than the self-connection of V5 (*Mean parameter estimate* = −0.03). This means that the (inhibitory) self-connection in the pSTS decreased more than the (inhibitory) self-connection in V5. In other words, since the (inhibitory) self-connection was decreased more towards zero, the pSTS activation is modulated by animacy. (B) VOIs used in the DCM analyses based on the mean of all participants' VOI centre coordinates and illustration of the modulatory connectivity between them. The first VOI, based on the peaks of the All Motion contrast, was in the MT+/V5 (44 −64 4; circled in blue). The other VOI was activated by the conjunction of the All motion contrast and the Animate – Inanimate motion contrast [All Motion & Animate – Inanimate motion] and was located in the pSTS (54 −50 16, circled in green). The colour of the line represents the source of the strongest bidirectional modulatory connection.

We created and estimated DCMs^24^ with DCM12 (version 5370) as implemented in SPM12b. The DCMs were based on the VOIs (volumes of interest) described above (V5 and the pSTS) and used the main effect of Animate – Inanimate motion to modulate the connections between these two regions (see Figure 2A). All DCMs were deterministic (as opposed to stochastic for DCMs without experimental input, see^25^), bilinear (as opposed to nonlinear DCMs, where activity between two regions is modulated by a third region, see^26^), two-state models^27^, with mean-centred inputs. Two-state DCMs differ from one-state models in that the activity in one brain region is modelled with both excitatory and inhibitory neuronal populations. This allows one to use positivity constraints that enforce extrinsic (between region) connectivity to be excitatory, while self or recurrent (intrinsic) connections are treated as inhibitory^27^. It is important to note that the hemodynamics in the current DCM are a function of excitatory states only – and the contributions to the BOLD signal from the inhibitory states are expressed indirectly, through interactions, with excitatory populations, at the neuronal level^27^. Note that the fixed and modulatory parameters were always scale parameters (exponentiated) to ensure positivity as per convention for two-state DCMs, so that the extrinsic connections were always excitatory^27^. Scale parameters of two-state DCMs are thus higher than parameter estimates from one-state DCMs. Our unexponentiated modulatory parameter estimates ranged from -2.7 to 3.9 Hz, similar to one-state DCM parameter estimates reported in other studies^11^,^28^. While the two state-DCMs use exponentiated scale parameters that introduce positivity constraints and are more plausible to interpret, these values are likely not normally distributed and heteroscedastic, because the exponential function is the inverse function of the natural logarithm (which is commonly used to transform data to meet the assumption of a normal distribution, see^29^,^30^). Thus, we used the original unexponentiated non-scale parameter estimates for all statistics, but the exponentiated parameter estimates for plots and interpretation.

### Post-hoc Bayesian model selection

Until recently, it was computationally expensive to estimate a large number of models with DCM^31^, especially with a large number of participants, as in the current study. A model space with n nodes has 2^n×n^ permutations of connections that can be turned on or off, which can be modulated by different experimental manipulations, leading to a combinatorial explosion^10^. We used a new method to find the model evidence for all possible models by only inverting (estimating) the full model^10^,^11^,^32^ as a prelude to identifying the best (reduced) model. This approach fits the full model – with all free parameters – to the data. The full model generally contains all possible intrinsic forward and backward connections, and all inputs and modulations of these connections by experimental factors. One then approximates the evidence for all possible reduced models, which have fewer parameters and are therefore nested within the full model. This is achieved by setting the prior variance over all combinations of free parameters (to zero). Based on the posterior density over the parameters of the full model, the approximate evidence for each reduced model can then be obtained using standard analytic results^10^,^11^. These post-hoc estimates of model evidence and the (conventional) free energy approximation (following inversion of reduced models) have been shown to yield very similar results with both simulated and real data^11^.

First, we used this post hoc model selection procedure^11^ to identify the best model out of all possible connection architectures with Bayesian model selection (BMS). Second, we looked at family-level inferences over all possible models showing whether fixed connections existed and whether they were modulated. This is done by computing a joint posterior probability density over parameter estimates for a group of participants, by using the posterior from one participant as the prior for the next participant, whose posterior then serves as the prior for the next participant, and so on^33^,^34^. The posterior probability is the probability that a model (or family of models) provides the best explanation for the measured data across participants^35^. The probabilities for all analyses were pooled in a fixed effects fashion, because we assumed that the underlying model structure did not vary across participants. The post-hoc optimisation also provides parameter estimates for individual participants that can be compared with conventional frequentist statistics^34^. Thus we present the simple average parameter estimates for the model with the highest evidence (the winning model) to elucidate the quantitative nature of the connection i.e. how much a connection is modulated^24^. The software implementation of the post-hoc optimisation for DCM can be found in the SPM function spm_dcm_post_hoc.m.

### Volume of Interest Selection

**Figure 3:**
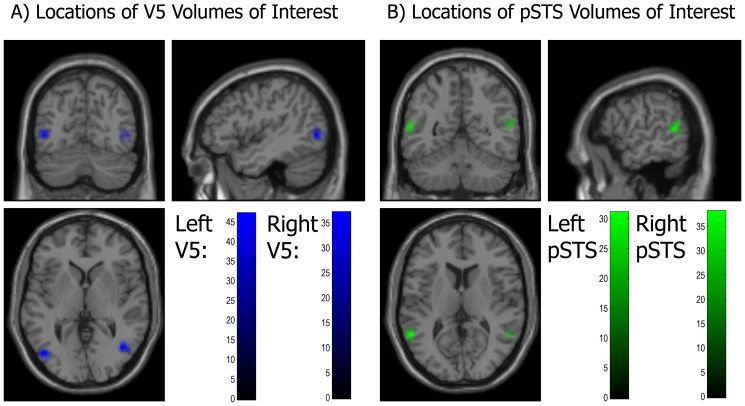
(A) Brain map showing the left and right V5 VOIs. The colour gradient bar indicates how many participants had the mean of their extracted voxels at a given location. (B) Brain map showing the left and right pSTS VOIs. The colour gradient bar indicates how many participants had the mean of their extracted voxels at a given location.

**Figure 4:**
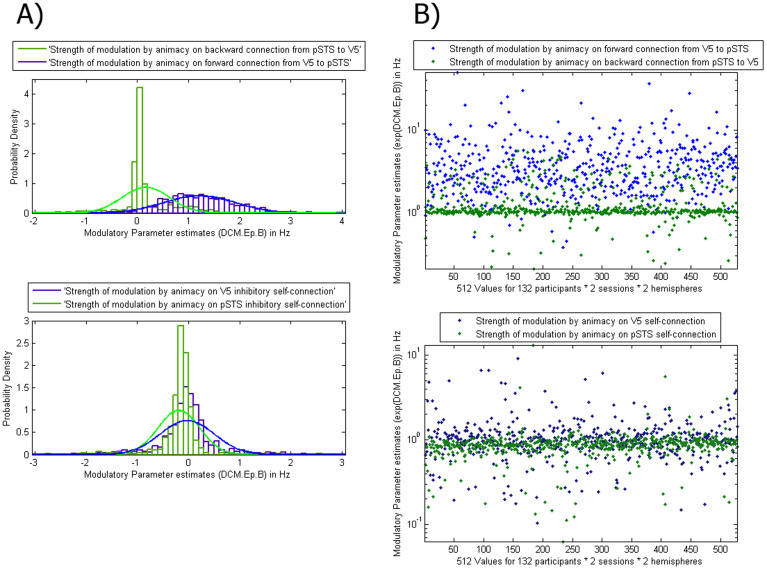
(A): Probability densities functions of parameter estimates for individual participants showed how strongly (self-)connections were modulated by animacy across participants, hemispheres and sessions. Upper Panel: The forward connection from V5 to the pSTS was more strongly modulated by animacy than the homologue backward connection. Lower Panel: The intrinsic self-connections of the pSTS was significantly lower than V5 and one can clearly see that the inhibitory self-connection in the pSTS has decreased towards zero consistently more than the inhibitory self-connection in V5. In other words, with the pSTS was no longer inhibited, this caused the pSTS activation observed in the Animate > Inanimate contrast. Figure 4 (B): Here the same data as in Figure 4A are plotted, showing all the different data points to highlight the consistency of the results.

To identify and summarise regional responses for further dynamic causal modelling we used standard procedures^37^. Timeseries from VOIs associated with the above contrasts were summarised using the SPM12b Eigenvariate toolbox: we extracted each participant's principal eigenvariate around the participant-specific local maxima activations nearest to the peak voxel of the group (between subject) GLM analysis (see Table 1 and 2). The radii of the VOI spheres were 6 mm and the search radii for local maxima from the group analysis were restricted to 20 mm. All voxels contributing to the eigenvariates were significant at *p* < 0.05 uncorrected and adjusted at *p* < 0.05 for the effects of interest (i.e. only for those regressors that were used in the DCMs for input or modulation). In order to replicate the results across sessions and hemispheres, we created separate DCMs for each hemisphere and each of the two sessions (four DCMs overall), which were then analysed together with repeated measures ANOVAs (see Figure 2B for a schematic of the model and Figure 4 for the results aggregated across participants, hemispheres and sessions). For each model, the first volume of interest (VOI) was based on maxima in the most active cluster of the All motion contrast (which was Animate and Inanimate motion over the implicit fixation baseline). These maxima were assigned by the SPM anatomy toolbox^38^ to MT+/V5 (sometimes called human occipital lobe area 5 (hOC5); right: 44 −64 4; left: −44 −74 4; see Table 1 and 2 for GLM results. Figure 3A and 3B show brain maps of the means of the extracted voxels of individual participants.). V5 was the most active region in our All Motion contrast, and has been shown to be highly sensitive to visual motion^12^. The second VOI was extracted at the local maxima of the most active clusters in each hemisphere based on the results of a conjunction analysis^39^,^40^. A conjunction of activations allows one to infer a co-occurrence of several effects in one area^40^: an activation map of a conjunction analysis will show those voxels as significant that would be significant in the two conjoined effects. The conjunction used here was the effect of the contrast [All Motion > Fixation Cross] & (logical AND) the contrast [Animate > Inanimate Motion] – i.e. [All motion > Fixation cross & Animate – inanimate motion]. The conjunction was performed to consider areas more active in Animate vs. Inanimate motion, but only in motion sensitive areas (activated by any type of motion). We used the more conservative test, testing against the conjunction null, instead of testing against the global null^40^. The second VOI was extracted from the pSTS (sometimes called inferior parietal cortex (IPC; more specifically PGa and PFm), right: 54 −50 16; left: −56 −52 10). The pSTS was highly active bilaterally: the peaks were local maxima in the most active cluster of each hemisphere with t-values above 8. The pSTS has been frequently implicated in animate motion processing (see discussion). Note that V5 was not significantly more active in this contrast, which might suggest that the stimuli were indeed well matched in terms of low-level motion properties. Finally, V5 and the pSTS have been shown to have strong (and reciprocal) anatomical connectivity^41^.

### Specification of dynamic causal models

Our particular interest was in the effect of animate motion processing on connections among sources in the distributed visual hierarchy. In particular, we wanted to know whether the effect of animate motion processing could be explained by changes – mediated by perceptual set (see for instance ref. 42) – in intrinsic and extrinsic connections. Furthermore, if these changes were in extrinsic connections, were they in the forward or backward connections? To answer these questions, we used Bayesian model comparison (BMS) of reduced models following inversion of a full model specified as follows: The full model comprised reciprocal connections between the motion sensitive area V5 and the pSTS. The driving input into the model – represented by the DCM.C matrix^24^– was the effect of All motion (Animate and Inanimate motion movement, modelled as a single regressor for both types of motion). This driving motion input entered either the posterior, lower region, V5 and modelled extrageniculate input, or the higher cortical node pSTS. Our hypothesis was that V5 would be the first region to show sensitivity to the presence of motion, and this would result in higher parameter estimates for V5 over the pSTS as the input region. V5 would subsequently influence activity in the pSTS region, but more so in the animate movement condition. These two cortical nodes were reciprocally coupled with extrinsic forward and backward connections, while intrinsic (self or recurrent) connections were treated as inhibitory. The effect of animacy was allowed to modulate all extrinsic and intrinsic connections. This full model was inverted for all participants and the resulting posterior densities over the connection strengths were used to perform family wise Bayesian model comparisons using post hoc optimisation. The models considered correspond to all possible combinations of the 10 free coupling parameters – corresponding to 2^∧^10 = 1024 reduced models. The 10 parameters comprised 2 fixed intrinsic parameters, 2 fixed extrinsic parameters, 4 parameters controlling the modulation of fixed connectivity and 2 parameters controlling the driving effect of All motion. To examine the connectivity in quantitative terms, we then analysed the posterior distribution over connections under the model with the highest evidence, using the distribution of estimates over participants.

In summary, the effect of perceptual set (animate motion) was allowed to change the intrinsic and extrinsic connectivity throughout the hierarchy. We then tested a series of reduced models comparing the evidence for (changes in) intrinsic connectivity, extrinsic connectivity or both. The evidence for these different hypotheses or models was assessed using a variational free energy approximation based upon the post hoc optimisation of reduced versions of the full model. Having identified the model with the greatest evidence, we then characterised the effects of motion and animate motion processing quantitatively, by examining the connection strengths and their bilinear modulation (the model can be replicated with scripts available online (https://github.com/HaukeHillebrandt/SPM_connectome) – also see discussion).

## Results

### Behavioural Results

Participants were able to judge whether motion was designed to be animate vs. inanimate as evidenced by the high accuracy levels of their responses (Correct responses: Animate condition: Session 1: *M* = 0.90, *SD* = 0.23, Session 2: *M* = 0.96, *SD* = 0.13; Inanimate condition Session 1: *M* = 0.83, *SD* = .21; Session 2: *M* = 0.84, *SD* = .26). However, a repeated measures ANOVA with factors session (1 vs. 2) and condition (animate vs. inanimate) showed that participants' responses became more accurate in session 2 (*F*(1,131) = 5.164, *p* < 0.025) and that they were more accurate in the animate condition compared with the inanimate condition (*F*(1,131) = 19.683, *p* < 0.001). The session × condition interaction was not significant (*F*(1,131) = 2.075, *p* < 0.152). Crucially, our DCM parameter estimates were consistent across sessions despite the modest behavioural differences between sessions (see Figure 4B).

### General Linear Model results

The All motion contrast (against a fixation cross as an implicit baseline) showed increased BOLD signal in many regions (whole-brain voxel-level FWE-corrected threshold of *p* < .05), likely due to very high power. In Table 1, we show the most significant activation with a t-value of 31 and above. The conjunction analysis of All Motion & Animate – Inanimate motion showed most activity in left and right middle temporal gyri (see Table 2).

### Dynamic Causal Modelling results

DCMs contained VOIs described above: V5 and the pSTS in the right (model 1) and left hemisphere (model 2). The post-hoc analysis (see Methods for details) finds the best model and furnishes the posterior probability of whether individual parameters exist or not. The latter is equivalent to family comparison, which tests whether a family of models with a certain parameter (e.g. a connection between two areas) has a higher probability than the family without this parameter^36^. Finally, we compared the strength of connections by examining the winning model's parameter estimates.

### Bayesian model selection

**Figure 5:**
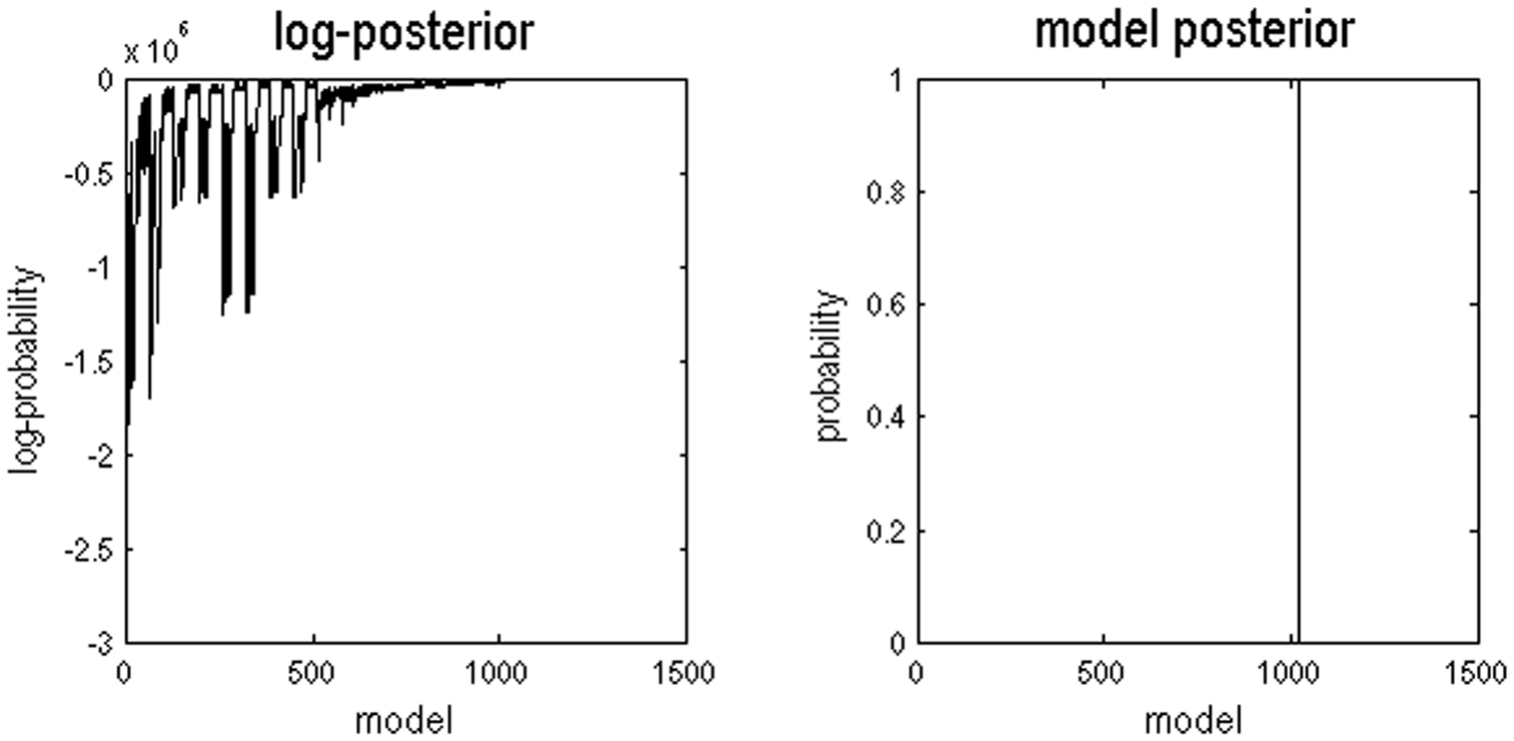
The left graph shows the range of log-posterior probabilities of all possible models examined. The right graph shows the posterior probabilities of all models. Model 1024 had the highest posterior probability of (almost) 1. This graph shows data for the first session and the right hemisphere, results for other sessions and hemispheres were similar.

We first assessed the model with the best evidence (a metric in which model fit is traded off against model complexity). Comparisons of the evidence for all possible 1024 models showed that the winning (optimal) model with the highest probability had a probability of (almost) 1 (Figure 5). The winning model in all four cases (2 (sessions) × 2 (hemispheres)) was always the full model that had all connections and all modulations (Figure 2A). This model has 10 free parameters describing the extrinsic and intrinsic connections and how these connections change with perceptual set. The profile of model (log) evidences over the ensuing 1024 models for one of the four cases is shown in Figure 5 (other plots were similar), suggesting that the full model had more evidence than any reduced variant (the probability was almost 1; the log-probability was almost 0). The next most probable model's probability was very low (almost 0; the log-probability was −70.9). The resulting Bayes factor, which can be obtained by dividing the winning model's probability (almost 1) by the next probable model's probability (almost 0), is considered decisive evidence for the winning model (corresponding to a highly significant difference)^43^. For comparison, even a Bayes Factor of 3:1 would still be considered positive evidence^43^. Additionally, this full connectivity was confirmed using several family-level inferences^33^, where families of models with certain parameters (such as connections or modulations existing) were compared with families of models without those parameters. This also showed that all (self) connections and their modulation by animacy were evident with a posterior probability of (almost) 1. The fact that we obtained strong evidence for all effects reflects the large sample size and high signal to noise ratio. Although there was very high evidence for the existence of all (self) connections and their modulations, this evidence cannot speak to the relative strength of forward versus backward connection (effect sizes). Therefore, we compared the relative strength of effective connectivity – under the winning model – in order to address specific hypotheses about the locus of animacy effects in quantitative terms.

### Comparison of connection strength

Since the full model had the highest evidence, we used its parameter estimates to test which (self) connections were modulated by animacy. We conducted two separate 2 (hemisphere: right, left) × 2 (session: 1,2) × 2 (connection type: forward V5-pSTS vs. backward pSTS-V5 connection or V5 self-connection, pSTS self-connection) repeated measures ANOVAs. These analyses of variance were applied to the modulatory (Animate – Inanimate) parameter estimates under the winning model from each participant. The results were clearly show a large and selective effect of connection type, which replicated across participants, hemispheres and sessions, such that in the first ANOVA, the forward connection from V5 to the pSTS was more strongly modulated by animacy (*Mean of log scaling parameter (unexponentiated)* = 1.22) than the homologue backward connection (*Mean of log scaling parameter (unexponentiated)* = 0.16; *F*(1,131) = 667.88, *p* < 0.001). In Figure 3 upper panel, we plot the associated scaling parameter (exponentiated) or modulation of these forward (V5-pSTS) (*Mean of scaling parameter (exponentiated)* = 4.374, 99% CI [3.799, 4.948]) and backward (pSTS-V5) connections (*Mean of scaling parameter (exponentiated)* = 1.326, 99% CI [1.221, 1.431]). The second ANOVA showed that the intrinsic self-connection of the pSTS (*Mean of log scaling parameter (unexponentiated)* = −0.19) was significantly lower than V5 (*Mean of log scaling parameter (unexponentiated)* = −0.03; *F*(1,131) = 27.47, *p* < 0.001). In Figure 3 lower panel, we plot the associated scaling parameter (exponentiated) or implicit modulation: there, one can clearly see that the inhibitory self-connection in the pSTS has decreased more (*Mean of scaling parameter (exponentiated)* = 0.90; 99% CI [.820, 0.977]) than the (inhibitory) self-connection in V5 (*Mean of scaling parameter (exponentiated)* = 1.13, 99% CI [1.024, 1.229]). In other words, the pSTS shows greater disinhibition during the animate condition. When applying the same analysis to the input parameter estimates, we found the input to V5 (*M* = 0.96, 99% CI [.872, 1.056]) was significantly higher than the input to the pSTS (*M* = −0.06, 99% CI [−.091, −.030]); *F*(1,131) = 817.08, *p* < 0.001), suggesting that V5 was the first area showing sensitivity to motion. Because all the above tests had a highly significant main effect of connection type, and parametric tests are generally robust to outliers^44^, we take these results as evidence for consistent effects across sessions, hemispheres and participants (see dot plots in Figure 4B). Finally, the 99.17% confidence intervals (CI) show that all parameter estimates differ significantly from zero (Bonferroni-corrected for multiple comparisons).

## Discussion

In the current large-scale Connectome fMRI study, we used DCM to examine effective connectivity between V5 and the pSTS in a task in which participants viewed animations of animate versus inanimate motion. We used a novel post-hoc model selection routine^10^,^11^ to investigate all possible dynamic causal models. Our results suggest that there was reciprocal fixed connectivity between V5 and the pSTS in both hemispheres, which was independent of the type of motion. The results also suggest that there was modulation of the forward and backward connection between V5 and the pSTS in both hemispheres by the animacy manipulation. Crucially, we found that the modulation of the forward connection was stronger than the modulation of the backward connection and that the inhibitory self-connection of the pSTS was decreased during the perception of animacy. In other words, since the inhibitory self-connection was decreased more towards zero by animacy, the pSTS was no longer inhibited, and thus activation was increased by animacy. Thus, our results show that motion selective areas (V5) influence, and were influenced by, a higher-level area (pSTS) responsive to motion trajectories of animate agents more than by movement of inanimate objects. It is important to note that the DCMs try to explain the activations and deactivations disclosed by standard SPM analyses. In other words, under the optimal DCM, differential responses of the pSTS are caused by modulation of intrinsic connectivity and the excitatory forward connection from V5.

Even though little is known about neural network interactions that support animacy perception, many functional localization studies implicate V5 in motion processing (for a review see ref. 12) and the pSTS in biological motion processing. The pSTS has been frequently shown to be involved in animate motion processing in neuroimaging studies (for a recent review, see ref. 45). This region's activity and structure also predict task performance on biological motion detection tasks^46^,^47^, and transcranial magnetic stimulation (TMS) over the pSTS disrupts the perception of biological motion^48^. Furthermore, the pSTS is active during attention to agentic movement^49^,^50^. For instance, Lee et al.^50^ showed that the pSTS activation (with a peak at: 56 −54 16; notably very close to our pSTS coordinates [right: 54 −50 16, left: −56 −52 10] resulting from our Animate – Inanimate motion contrast) was more active when participants were instructed to attend to whether dots chased each other (animate condition) as opposed to whether dots were making mirror movements (inanimate condition) in statistically identical random motion.

Our findings can be interpreted in the framework of predictive coding as an emerging view of localization of brain function that is based on context and prediction; a view that is becoming increasingly popular in social neuroscience^51^,^52^. In predictive coding, forward, bottom-up connections have been hypothesized to propagate prediction error signals about (unexpected) sensory information associated with the stimulus from ‘lower' (sensory) brain areas to areas that are ‘higher' in the cortical hierarchy^53^,^54^. Top-down, backward connections then send predictions based on an internal generative model about the stimulus to lower sensory areas to minimize sensory prediction error (see ref. 5, 53). Furthermore, the selective modulation of prediction errors provides a mechanism for selectively attending to particular prediction errors that inform high-level representations^15^. One recent study showed that animate motion captures attention and is responded to faster than inanimate motion^4^. In Lee et al's^50^ and our study, the lower level properties (such as direction changes) of the motion were completely matched, but participants' prior expectation to attend more to the animate motion^55^ was associated with more activity in the pSTS, possibly because participants' internal model cannot predict animate motion as well. This is also supported by a study showing neural adaptation to repeated exposure of the same animate motion trajectories^56^. Another study used eye-tracking, while participants viewed the same stimuli used in our study, and showed that participants fixated longer on animate than inanimate stimuli, suggesting that they are indeed harder to predict^55^. This increased attention to the motion might be interpreted as more frequent updating of the internal model for the motion trajectory of the triangles, because the initial predictions about animate stimuli break down more rapidly (more prediction errors) than predictions about inanimate motion trajectories.

### Future research facilitated by open science

Recently, fMRI studies have been criticized for low statistical power due to relatively small sample sizes^57^, (but see ref. 58). A recent study reported that fMRI studies with small sample sizes discover as many foci as larger studies, even though more foci should be activated as sample size and thus statistical power increases^59^. This is suggestive of a strong reporting bias in the fMRI literature, leading David and colleagues to call for the generation of standardized large-scale evidence in the field^59^. Here we analysed data from 132 participants to achieve high statistical power and showed consistent results that replicated across participants, sessions and hemispheres.

Open sharing of task-based fMRI data is becoming increasingly popular and has a wide array of advantages^60^,^61^. The HCP data are publically accessible, and the analysis scripts used in the current study are freely available online (https://github.com/HaukeHillebrandt/SPM_connectome), which will facilitate replications and extensions of the present findings^62^. As more data become available, the analyses can be extended to more participants. Moreover, as the HCP provides data from other imaging modalities^16^, these can be incorporated into existing models. For instance, DCMs might be improved with tractography-based^63^ or electrophysiological priors^64^. It would be interesting to see whether future DCM extensions can improve the model fit: for instance, nonlinear DCMs where one region can influence connections between other regions, can sometimes outperform bilinear DCMs^26^. Future studies could compete in a challenge to find a plausible model that best explains the same data, as is sometimes done in machine learning.

Our study is limited by the assumptions upon which the methods used here are based. For a critical review on these biophysical and statistical foundations of DCM see Daunizeau et al.^65^ and for a review of fMRI as a method see Logothetis^66^.

### Conclusion

Here, we used DCM to provide evidence from data from a very large number of participants for set-dependent changes in the sensitivity of the pSTS to both forward afferents from motion sensitive area V5 and recurrent connections within the pSTS. These were likely to be mediated by top-down effects that establish the perceptual set that was engaged during the perception of animate motion. Furthermore, these results speak to the reproducibility and consistency of effective connectivity estimates in a large number of participants and demonstrate the increase in statistical efficiency afforded by large cohorts. Our results show that, while both forward and backward connections from V5 to the pSTS were modulated when participants view animate vs. inanimate movement, the forward connection from V5 to the pSTS was more strongly modulated than the backward homologue. This suggests that the biological complexity of modelling and predicting movement of other agents leads to higher sensory prediction error.

## Author Contributions

H.H., K.J.F. and S.J.B. designed the study, interpreted the data and wrote the paper. H.H. performed the data analysis and made the figures.

## Figures and Tables

**Table 1:** GLM results. All motion over implicit baseline contrast

Maximum:	T-value:	MNI coordinates:			Region:	SPM anatomy toolbox assignment:	Probability:
1	33.18	32	−70	28	Right Middle Occipital Gyrus		
2	32.41	−42	−68	2	Left Middle Occipital Gyrus	left hOC5 (V5)	20%
3	31.97	−26	−74	26	Left Middle Occipital Gyrus		
**4**	**31.78**	**44**	**−64**	**4**	**Right Middle Temporal Gyrus**	**right hOC5 (V5)**	40%
**5**	**31.34**	**−44**	**−74**	**4**	**Left Middle Occipital Gyrus**	**left hOC5 (V5)**	50%

The analyses were performed with FWE correction at *p* < 0.05, whole brain level and cluster size of more than 5 voxels. Listed are only those peaks that have a t-value of 31 or above.

Coordinates used for VOI extraction are in BOLD.

**Table 2:** GLM results. Conjunction analysis of All motion & Animate – Inanimate motion

**Cluster 1 (2405 voxel): Left hemisphere**							
Maximum:	T-value:	MNI coordinates:			Region:	SPM anatomy toolbox assignment:	Probability:
1	16.71	−20	−76	−36	Left Cerebellum	Left Lobule VIIa Crus II (Hem)	
						Lobule VIIa Crus II (Hem)	74%
						Lobule VIIa Crus I (Hem)	22%
2	13.51	−20	−72	−28	Left Cerebellum	Left Lobule VIIa Crus I (Hem)	
						Lobule VIIa Crus I (Hem)	50%
						Lobule VI (Hem)	50%
3	11.75	−42	−50	−14	Left Fusiform Gyrus		
4	10.45	−46	−56	−16	Left Fusiform Gyrus		
5	10.32	−30	−46	−6	Left Lingual Gyrus		
6	10.15	−32	−46	−22	Left Fusiform Gyrus	Lobule VI (Hem)	2%
7	9.91	−34	−54	−18	Left Fusiform Gyrus		
8	9.34	−32	−42	−10	Left ParaHippocampal Gyrus		
9	9.32	−52	−26	−2	Left Middle Temporal Gyrus		
10	9.16	−52	−36	2	Left Middle Temporal Gyrus		
11	8.99	−30	−40	−22	Left Fusiform Gyrus		
12	8.97	−24	−48	−10	Left Lingual Gyrus		
13	8.89	−52	−52	22	Left Middle Temporal Gyrus		
14	8.62	−32	−64	−24	Left Cerebellum		
**15**	**7.82**	**−56**	**−52**	**10**	**Left Middle Temporal Gyrus (pSTS)**		
**Cluster 2 (1118 voxel): Right hemisphere**							
Maximum:	T-value:	MNI coordinates:			Region:	SPM anatomy toolbox assignment:	Probability:
1	14.44	48	−24	−6	N/A		
2	13.1	48	−36	4	Right Middle Temporal Gyrus		
3	10.81	52	4	−18	Right Medial Temporal Pole		
4	10.53	58	−40	6	Right Middle Temporal Gyrus	IPC (PGa)	10%
5	9.73	56	−32	−2	Right Middle Temporal Gyrus		
6	9	50	−4	−16	Right Middle Temporal Gyrus		
**7**	**8.72**	**54**	**−50**	**16**	**Right Middle Temporal Gyrus**	**IPC (PGa)**	**30%**
						**IPC (PFm)**	**20%**

The analyses were performed with FWE correction at *p* < 0.05, whole brain level and cluster size of more than 5 voxels.

Listed are only those peaks that have a t-value higher than the pSTS that were extracted in both clusters.

Coordinates used for VOI extraction are in BOLD.
